# Resource-Based Internet Intervention (Med-Stress) to Improve Well-Being Among Medical Professionals: Randomized Controlled Trial

**DOI:** 10.2196/21445

**Published:** 2021-01-11

**Authors:** Ewelina Smoktunowicz, Magdalena Lesnierowska, Per Carlbring, Gerhard Andersson, Roman Cieslak

**Affiliations:** 1 Department of Psychology SWPS University of Social Sciences and Humanities Warsaw Poland; 2 Department of Psychology Stockholm University Stockholm Sweden; 3 Department of Behavioural Sciences and Learning Linköping University Linköping Sweden; 4 Department of Clinical Neuroscience Karolinska Institutet Stockholm Sweden; 5 Trauma, Health, and Hazards Center University of Colorado Colorado Springs Colorado Springs, CO United States

**Keywords:** well-being, job stress, internet intervention, self-efficacy, perceived social support, medical professionals

## Abstract

**Background:**

Medical professionals are exposed to multiple and often excessive demands in their work environment. Low-intensity internet interventions allow them to benefit from psychological support even when institutional help is not available. Focusing on enhancing psychological resources—self-efficacy and perceived social support—makes an intervention relevant for various occupations within the medical profession. Previously, these resources were found to operate both individually or sequentially with self-efficacy either preceding social support (cultivation process) or following it (enabling process).

**Objective:**

The objective of this randomized controlled trial is to compare the efficacy of 4 variants of Med-Stress, a self-guided internet intervention that aims to improve the multifaceted well-being of medical professionals.

**Methods:**

This study was conducted before the COVID-19 pandemic. Participants (N=1240) were recruited mainly via media campaigns and social media targeted ads. They were assigned to 1 of the following 4 groups: experimental condition reflecting the cultivation process, experimental condition reflecting the enabling process, active comparator enhancing only self-efficacy, and active comparator enhancing only perceived social support. Outcomes included 5 facets of well-being: job stress, job burnout, work engagement, depression, and job-related traumatic stress. Measurements were taken on the web at baseline (time 1), immediately after intervention (time 2), and at a 6-month follow-up (time 3). To analyze the data, linear mixed effects models were used on the intention-to-treat sample. The trial was partially blinded as the information about the duration of the trial, which was different for experimental and control conditions, was public.

**Results:**

At time 2, job stress was lower in the condition reflecting the cultivation process than in the one enhancing social support only (*d*=−0.21), and at time 3, participants in that experimental condition reported the lowest job stress when compared with all 3 remaining study groups (*ds* between −0.24 and −0.41). For job-related traumatic stress, we found a significant difference between study groups only at time 3: stress was lower in the experimental condition in which self-efficacy was enhanced first than in the active comparator enhancing solely social support (*d*=−0.24). The same result was found for work engagement (*d*=−0.20), which means that it was lower in exactly the same condition that was found beneficial for stress relief. There were no differences between study conditions for burnout and depression neither at time 2 nor at time 3. There was a high dropout in the study (1023/1240, 82.50% at posttest), reflecting the pragmatic nature of this trial.

**Conclusions:**

The Med-Stress internet intervention improves some components of well-being—most notably job stress—when activities are completed in a specific sequence. The decrease in work engagement could support the notion of dark side of this phenomenon, but further research is needed.

**Trial Registration:**

ClinicalTrials.gov NCT03475290; https://clinicaltrials.gov/ct2/show/NCT03475290

**International Registered Report Identifier (IRRID):**

RR2-10.1186/s13063-019-3401-9

## Introduction

### Background

Well-being at work has been the focus of numerous workplace interventions, as it is associated with important outcomes both for the organization and the individual, including performance [1], absenteeism [2], and turnover intentions [3]. One occupational group that is particularly at risk of losses in terms of well-being is health care professionals [4-6]. In this study, we tested the efficacy of an internet intervention that aims to enhance psychological resources—perceived social support and self-efficacy—to improve the well-being of medical professionals. As this study was conducted before the outbreak of the COVID-19 pandemic in 2020, we managed to capture the everyday functioning of medical professionals.

Regardless of occupation, workplace interventions represent various approaches. Some of them are contextual and focus on factors (job demands and resources) that are specific to a given organization [7,8], whereas others are context-free because they target personal characteristics, such as self-efficacy, hope, and optimism [8]. Finally, there are psychotherapies [9,10] that focus on specific mental health disorders, such as depression and anxiety, albeit in the context of work.

Although many of these interventions have been found to be effective, their accessibility is usually constrained, and they can be difficult and costly to scale-up. Technology overcomes some of these limitations. Over the past three decades, internet interventions have been shown to deliver effective treatments for a variety of mental health problems [11-13]. More recently, their efficacy has also been tested in work environments with promising results [14,15]. Some of these interventions have been conceptualized within the cognitive behavioral therapy (CBT) framework [16], whereas others employed mindfulness [17] or stress management techniques [18]. However, although some programs [19] were based on theoretical models such as the stress and cognitive appraisal model by Lazarus and Folkman [20], they rarely test frameworks that have been specifically developed in the context of occupational health (eg, job demands-resources model [21]) or adapted to it (eg, conservation of resources theory [COR] [22]).

COR [22] originated as a conceptual framework of general stress but has since repeatedly served as a backbone for research on occupational well-being [23,24]. One of its fundamental premises is the resource investment principle, also known as a gain spiral. It refers to a process in which people who possess certain resources (eg, time) invest them to generate new ones (eg, learn new skills) and ultimately lower their risk of future losses. This process has already been empirically demonstrated [25-27]; however, studies have tended to focus on contextual job resources and have rarely been experimental (with notable exceptions [28]). Meanwhile, personal resources are by definition context-free. A risk of focusing on them in an intervention is that they are difficult to customize to the unique combination of demands and resources in a given workplace. However, at the same time, such an intervention has the advantage of not being limited to only a few workplaces but is applicable across organizations.

Resources that have repeatedly been shown to be associated with well-being at work are social support and self-efficacy. Social support is usually defined in relation to its source (eg, from supervisor, friends, or family), and within those relationships, it can be either received or provided [29]. Social support that does not refer to the outside source is a perceived one and is defined as one’s belief that help will be provided when needed [30], making it similar to optimism and hope [31]. Moreover, the relationship between received and perceived support is only moderate [31], showing that they are distinct concepts. Therefore, it is justified to enhance perceived social support independently from a specific social context. Finally, social support is a process that generates new resources [32], which makes it uniquely suitable as a chain in the gain spiral. Meanwhile, self-efficacy, another key resource, is defined as “beliefs in one’s capabilities to organize and execute the courses of action required to produce given attainments” [33]. Its 4 major sources have been identified as mastery and vicarious experiences, persuasion, and reinterpretation of emotional and physiological states [33], with the first considered the most powerful [34]. As self-efficacy is more predictive of a given outcome when it is task- or domain-specific [35], it is more beneficial to enhance contextual self-efficacy in a workplace intervention (eg, related to coping with job stress) than a general one.

Hobfoll et al [32] suggested that people abundant in personal resources such as self-efficacy are in a better position to elicit support from others but also that this relationship works in reverse, and social support can prevent the depletion of personal resources. Called the cultivation and enabling hypotheses, this two-way relationship was later conceptualized in the form of 2 arguments [36]. According to the former, people with higher self-efficacy are more likely to reach out and solicit social support when needed, whereas the latter suggests that using members of a social network as models—in particular those that are similar and/or face similar situations—or benefiting from their verbal assurance can increase the sense of efficacy. Not only theoretical but also empirical support was found for both propositions, however mostly in the context of health [37] and traumatic stress [38,39] but not for outcomes specific to work. In addition, although many of the studies were longitudinal, few were of experimental design. Therefore, in this study, we aim to verify the efficacy of enhancing social support and self-efficacy to improve well-being in a randomized controlled trial (RCT) and test whether the sequence in which they are strengthened is important.

The results of the sixth European Working Conditions Survey show that people working in the health sector experience the highest level of work intensity [40]. Their well-being impacts the services they provide as well as the functioning of health care systems [41]. Thus, improving it is important not only for themselves but also for the patients and their entire workplace communities.

The term well-being covers numerous occupational phenomena. In this intervention, we focus on 5 that are prevalent in the health care setting: job stress, job burnout, work engagement, job-related traumatic stress, and depression. Our primary focus is on job stress and burnout. Stress is a response to a situation when resources are not available or are insufficient to offset demands that are placed upon employees [42], whereas job burnout, which has recently been added to the 11th Revision of the International Classification of Diseases [43], is defined as “a prolonged response to chronic emotional and interpersonal stressors on the job” [44]. Depending on the conceptualization, it comprises 1, 2, or 3 dimensions [45]. Although it is difficult to assess the prevalence of occupational issues such as job stress and job burnout among medical professionals, there is evidence to show that these problems are encountered globally, including in the cultural West [5,46], sub-Saharan Africa [47], and Japan [48]. A recent systematic review that extracted data from populations of 45 countries reported that up to 80.5% of physicians experienced burnout [49], making this a problem that needs to be addressed urgently.

Work engagement is defined as “a positive, fulfilling, work-related state of mind that is characterized by vigor, dedication, and absorption” [50]. Although it is negatively related to job burnout, they are not opposite poles of one dimension. Recent findings show that employees exhibit different burnout-engagement profiles: from those representing clear discrepancies (ie, high engagement with low burnout and vice versa) to profiles showing that people can be low, moderate, or high on both these dimensions at the same time [51]. Thus, interventions that target job burnout should also measure their impact on work engagement.

Job-related traumatic stress is another outcome that belongs to the occupational context, albeit not to all professions. Defined as stress resulting particularly from indirect exposure to aversive details of traumatic events via face-to-face contact with traumatized individuals or exposure to drastic materials [52], it has been widely recognized as a significant occupational burden among health professionals that needs addressing [53,54].

Contrary to previous outcomes, depression is not a typical occupational phenomenon. However, it is an important component of work-related well-being: it is prevalent across professions and has been found to predict poor job performance [55] and sickness absences [56]. Moreover, although criteria distinguishing depression from job burnout are defined, it remains problematic to differentiate them [57].

### Objectives

The main goal of this study was to test whether a Med-Stress internet intervention dedicated to medical professionals would be effective in reducing job stress, burnout, depression, and job-related secondary stress and in increasing work engagement through the enhancement of 2 psychological resources: perceived social support and self-efficacy. We compared 2 experimental conditions, in which self-efficacy and social support were enhanced sequentially, with 2 active controls, in which only self-efficacy or only social support was targeted. We expected that experimental conditions, which are designed to build 2 resources, would prove to be more beneficial than active control ones—that target only a single resource—in terms of reducing negative outcomes (ie, job stress, burnout, depression, and job-related secondary stress) and improving a positive one (ie, work engagement) both at posttest and at a 6-month follow-up. Furthermore, we aim to test whether one sequence of resources—in line with either the enabling or cultivation hypothesis—would turn out to be more beneficial than the other.

## Methods

### Study Design

The Med-Stress study was designed as a 4-arm parallel RCT comparing the posttest and follow-up effects of 2 experimental and 2 active control conditions. The trial was conducted on web. This study was approved by the Ethical Review Board at SWPS University of Social Sciences and Humanities (opinion 4/2018 issued on February 13, 2018) and registered on ClinicalTrials.gov (NCT03475290). The study protocol has been previously published [58].

### Recruitment and Study Participants

Participants were recruited between October 2018 and April 2019 via targeted social media campaigns, advertisements via radio and press, professional forums, and through medical organizations. The following inclusion criteria were applied: (1) being at least 18 years old and (2) representing the health-related profession that involved direct patient care. A total of 1575 people were registered for the study; however, 335 did not complete the required baseline assessment. The final sample size was N=1240.

### Power Calculation

To ensure a statistical power of 0.90 to detect the posttest effect of comparisons between study conditions, we conducted an a priori sample size estimation using G*Power 3.1 3.1 [59]. Taking into account the previous studies on the effectiveness of internet interventions for general and job-related stress [15,60], we aimed to detect the minimum effect sizes of *d*=0.40 for the comparisons between conditions at 2 measurement points while controlling for baseline scores at an alpha error level of .002, reduced to correct for multiple comparisons (ie, 5 between-group comparisons and 5 outcomes). A power analysis showed that a sample of 607 was needed. As we expected a high dropout rate, we aimed to recruit a sample of 1200. As the analyses were ultimately conducted using linear mixed effects models (*see the Statistical Analysis* section for more details), we conducted an additional analysis using the powerlmm package in R [61] to find that with the sample of 1240 participants, we had the power to detect an effect size of approximately *d*=0.21 in pairwise comparisons. The dropout rate was higher than expected (1023/1240, 82.50%), and thus, we did not have enough power to test for robustness of effects in the per protocol analysis.

### Procedure

**Figure 1:**
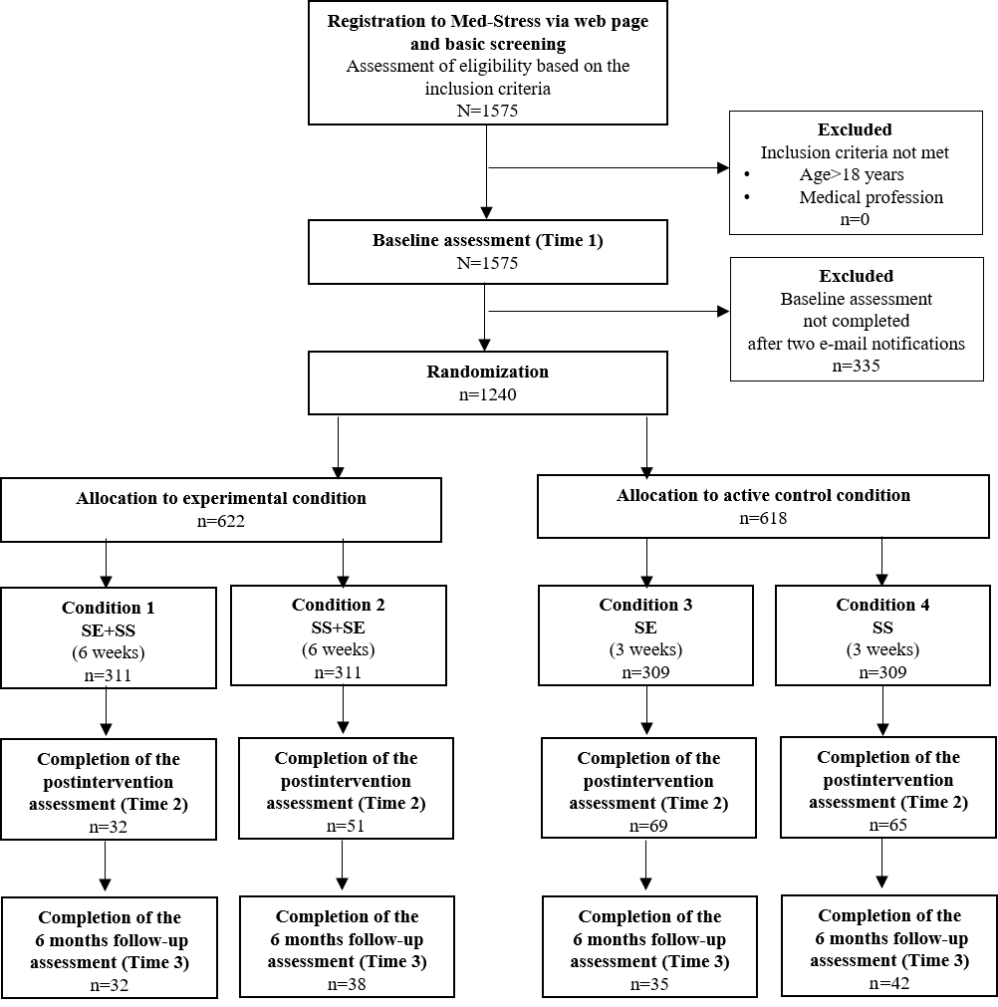
Flow of participants. SE: self-efficacy enhancement module; SE+SS: self-efficacy and perceived social support sequential enhancement module; SS: perceived social support enhancement module; SS+SE: perceived social support and self-efficacy sequential enhancement module.

The study flow is presented in Figure 1. Participants were directed to the Med-Stress website, where they filled out a screening to ensure they met the inclusion criteria. The registration process was finalized when they signed a web-based informed consent form. Subsequently, participants were asked to fill out a baseline (time 1) assessment. Only those who completed this step were randomized to 1 of the 4 conditions. The intervention lasted either 6 weeks (experimental conditions) or 3 weeks (active controls). Activities in the intervention were released once a week for a participant. Although we encouraged participants to complete all tasks, this was not a prerequisite to access subsequent exercises. Immediately after completing the intervention (at the posttest, time 2) and 6 months after the baseline assessment (follow-up, time 3), participants were invited to fill out the questionnaires on web. Each time, participants received 3 email remainders (2 automatic and 1 personalized).

### Intervention

**Figure 2:**
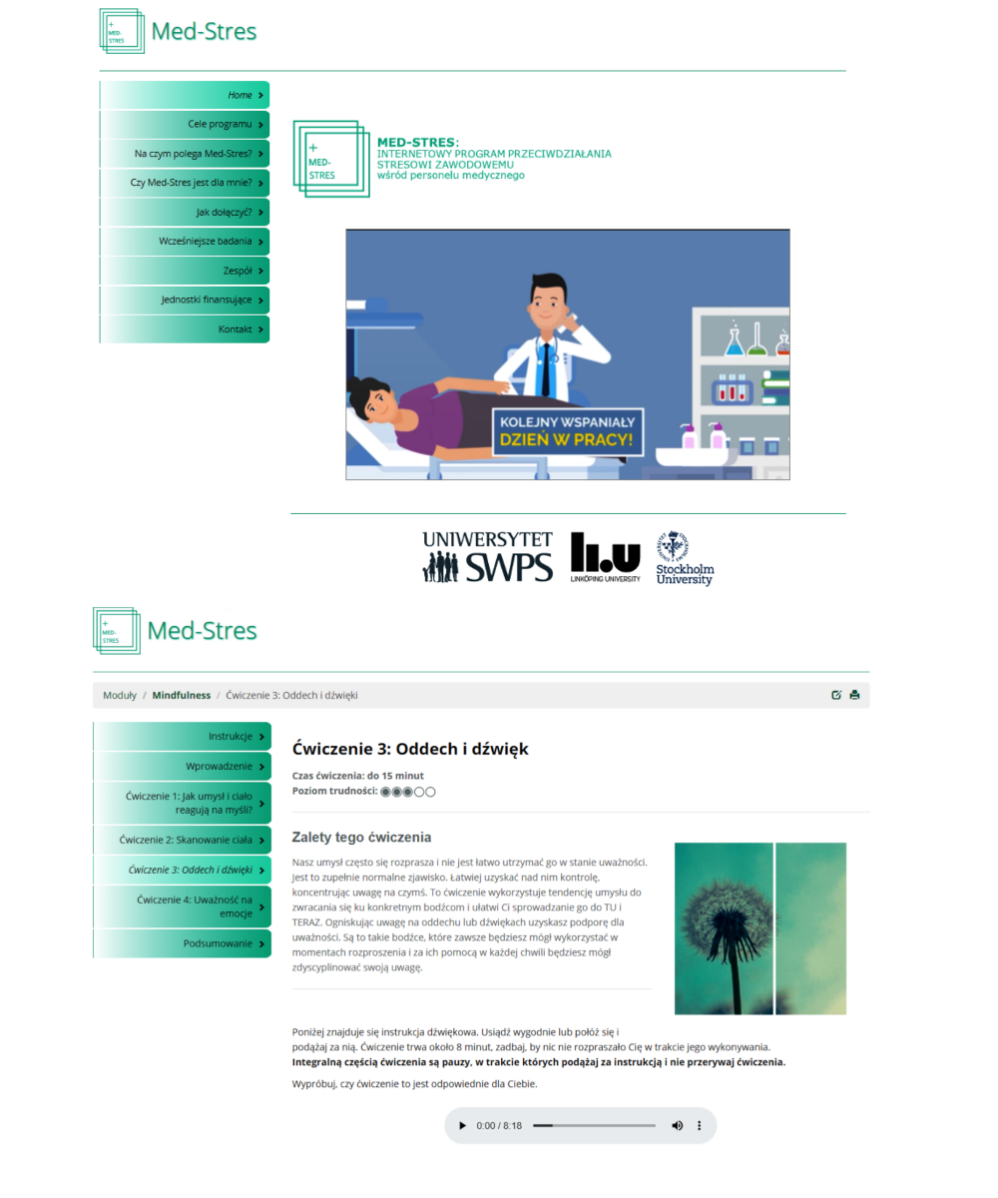
Med-Stress internet intervention: (a) mastery experience and (b) mindfulness exercises.

Med-Stress is a self-guided internet intervention. It contains 2 main modules that were made available to participants in different variants depending on randomization to study conditions: (1) self-efficacy and perceived social support sequential enhancement modules (SE+SS; experimental condition), (2) perceived social support and self-efficacy sequential enhancement modules (SS+SE; experimental condition), (3) self-efficacy enhancement module (SE; active control condition), and (4) perceived social support enhancement module (SS; active control condition). There were 3 exercises per module. Each consisted of psychoeducational animated clips and interactive tasks requiring both web-based and offline activities. To complete all tasks within each exercise, participants needed up to 1.5 hours. In addition, everyone had access to 4 modules: relaxation, mindfulness, cognitive restructuring, and lifestyle, which were optional and available throughout the intervention. Such a design allowed the participants to partially self-tailor the intervention. Exercises in each module were evidence based and developed in a participatory manner, as a result of the preimplementation study among 744 medical professionals conducted at the Med-Stress developmental stage (Lesnierowska et al, unpublished data, 2018). Exercise descriptions are presented in Table 1, and the example screenshots are shown in Figure 2. The program was run on the Iterapi platform developed at the Department of Behavioral Sciences and Learning at Linköping University, Sweden [62].

**Table 1:** The content of the Med-Stress intervention: obligatory and optional modules.

Modules		Exercises	Aims
**Obligatory**			
	Self-efficacy enhancement	Mastery experience Vicarious experience Action planning	To enhance self-efficacy via recollecting past successes in coping with job stress, taking inspiration from other people (models), and creating a coping plan, based on the “if-then” approach to address potential coping barriers. The exercises are released weekly.
	Perceived social support enhancement	Received support and cognitive distortions Social skills and peer support Action planning	To enhance perceived social support through verifying distorted assumptions about obtaining support, recalling past situations when the actual support was received, engaging into real receive-provide support interactions with other participants (chatrooms), and creating a coping plan, based on the “if-then*”* approach aimed at obtaining social support. The exercises are released weekly.
**Optional**			
	Relaxation	Breathing Progressive relaxation Visualizing the body’s warmth and weight Visualizing a calm place	To support the coping process by stress relief techniques and actions. The exercises in this module aim at active relaxation. The exercises are available during the whole intervention.
	Mindfulness	How do body and mind react to thoughts? Body scanning Breathing and sounds Being mindful of emotions	To support the coping process by stress relief techniques and actions. The exercises in this module aim at deliberate attention on a present experience. The exercises are available during the whole intervention
	Cognitive restructuring	Opinion or fact Identifying thinking traps How important will this be in the future?	To support the coping process by stress relief techniques and actions. The exercises in this module aim at skills such as distinguishing opinions from facts or identifying cognitive traps. The exercises are available during the whole intervention
	Lifestyle	Physical activity Pleasant activities	To support the coping process by stress relief techniques and actions. The exercises in this module aim at implementing physical activity or pleasant activities that foster stress reduction. The exercises are available during the whole intervention

### Randomization Procedure

Participants were automatically allotted to 1 of the 4 study conditions in line with the predefined randomization protocol generated via randomizing software (randomizer.org, a free service offered by Social Psychology Network). We applied block randomization with a block size of 4. The allocation ratio was 1:1:1:1 to ensure an equal number of participants in each study condition. Masking was partially achieved: allocation was not revealed to the participants; however, they had previously been informed that the intervention would last 3 or 6 weeks depending on the intervention variant.

### Measures

#### Primary Outcomes

##### Job Stress

Job stress was assessed using the 14-item Perceived Stress Scale [42]. Instructions were adapted for the occupational context. The questionnaire includes 14 items describing the range of stress symptoms rated from 0 (never) to 4 (very often). Cronbach alphas were .85 at time 1, .86 at time 2, and .89 at time 3.

##### Job Burnout

Job burnout was measured using the Oldenburg Burnout Inventory [63]. The scale consists of 16 items. Participants were asked to respond on a scale ranging from 1 (strongly agree) to 4 (strongly disagree). The reliability of the scale was α=.87 at time 1, α=.89 at time 2, and α=.91 at time 3.

#### Secondary Outcomes

##### Depression

Depression was assessed using the Patient Health Questionnaire-9 [64]. The scale has 9 items with responses ranging from 0 (not at all) to 3 (nearly every day). Cronbach alphas were α=.87 at time 1, α=.88 at time 2, and α=.89 at time 3.

##### Work Engagement

Work engagement was measured using the Utrecht Work Engagement Scale-3 [65]. This shortened tool includes 3 items with a response scale ranging from 0 (never) to 6 (always). The Cronbach alphas were .73 at time 1, .78 at time 2, and .83 at time 3.

##### Job-Related Traumatic Stress

Job-related traumatic stress was assessed using the Posttraumatic Stress Disorder Checklist 5 [66] with modified instructions. It consists of 20 items. Participants assessed the severity of the symptoms on a scale ranging from 0 (not at all) to 4 (extremely). The Cronbach alphas were α=.95 at time 1, α=.96 at time 2, and α=.96 at time 3.

#### Other Measures

##### Self-Efficacy to Manage Job Burnout and Work Stress

Self-efficacy to manage job burnout and work stress was measured using the Work Stress and Job Burnout Self-Efficacy Scale [67]. The scale has 28 items rated from 1 (I am definitely not capable) to 7 (I am definitely capable). The Cronbach alphas were .93 at time 1 and .96 at both time 2 and time 3.

##### Social Support Self-Efficacy

Social support self-efficacy was measured using the modified version of the Berlin Social Support Scales, Subscale 3 [68]. The tool was adapted to the context of social support self-efficacy. It consists of 5 items with a response scale ranging from 1 (I am definitely not capable) to 7 (I am definitely capable). The reliability was α=.88 at time 1, α=.92 at time 2, and α=.83 at time 3.

##### Perceived Social Support

Perceived social support was assessed using the Who Can You Count On Scale [69]. The questionnaire consists of 32 items with a response scale ranging from 1 (to very little extent) to 5 (to a great extent). The Cronbach alphas were .95 at time 1 and .96 at time 2 and time 3.

##### Secondary Trauma Exposure

Secondary trauma exposure was measured using The Secondary Trauma Exposure Scale [70]. Participants indicated their answers on a yes or no scale in response to whether they indirectly experienced each of the 10 listed traumatic events. The volume, frequency, and ratio of exposure were assessed using a single item with response scales ranging from 1 (none or never) to 7 (a few thousands or every day), and from 0% to 100% for the exposure ratio. Due to scale construction, the Cronbach coefficient was not computed.

##### Expectancy of Improvement and Perceived Credibility of the Intervention

Expectancy of improvement and perceived credibility of the intervention were assessed using the Credibility and Expectancy Questionnaire Version II [71]. The scale consists of 6 items representing 2 subscales: perceived intervention credibility and expectancy of improvement. Responses ranged from 1 (not at all) to 9 (very). The reliability for the perceived intervention credibility subscale was α=.81 and for the expectancy of improvement was α=.89.

##### Intervention Usage

We assessed the use of obligatory and optional modules. For the former, engaging in at least one weekly assigned task was coded as activity. Optional modules contained a number of similar exercises that participants could choose from, thus engaging in at least one of them was coded as completion.

#### Statistical Analysis

Analysis of variance (ANOVA) for continuous and chi-square tests for categorical data were used to conduct a randomization check and dropout analysis. We did not formulate predictions regarding differences in resources (ie, perceived social support and self-efficacy) between study conditions, but we did expect them to increase between time 1 and time 2 and to remain stable by time 3. To test the change over time, we applied linear mixed effects models with a restricted maximum likelihood estimation, an unstructured covariance matrix, and time as a fixed factor compared between 3 measurement points.

To verify the effect of intervention conditions on the 5 outcome variables over the course of 3 time points, we applied linear mixed effects models with restricted maximum likelihood estimation, unstructured covariance matrices of random effects, and random slopes and intercepts [72], separately for each of the 5 outcomes. Time variable was coded as time 1=0 (baseline), time 2=1 (posttest), and time 3=2 (follow-up). To make comparisons between each active control group and experimental group as well as between 2 experimental conditions, we created 3 dummy variables in which 2 controls and 1 experimental condition subsequently served as a reference category. It is important to note that our research question was not about the differences in changes over time between groups but about the differences in the outcomes between study conditions at 2 time points: the posttest and at the follow-up. This was reflected in the study protocol [58] where we planned to conduct separate analyses at 2 measurement points. Ultimately, to take into account the dependency between multiple measurements, we used linear mixed effects models. For each outcome, we applied a top-down approach by first fitting a loaded model with fixed and random effects, and we subsequently compared it with 2 nested models: one without random slope and one without random intercept. In the case of job-related secondary traumatic stress, we additionally controlled for exposure to traumatic events, which is a critical factor for the development of posttraumatic symptoms [52]. Finally, we tested whether there were predictors of the 2 primary outcomes (job stress and burnout). To do so, we first calculated the residual gains by subtracting the standardized initial score (Z1) multiplied by the correlation between the initial and posttreatment scores from the standardized posttreatment score (Z2) [73]. Next, we inverted the gains’ values so that positive scores reflected reduction. These residual gains were entered separately as outcome variables in the regression analyses performed with 10,000 bootstrapped replications. All analyses in this study were conducted on the intention-to-treat sample. The multiple imputation (MI) procedure was applied using 10 iterations. All baseline scores, available posttest and follow-up scores, and all variables that were found to differentiate completers and dropouts (see below in *Preliminary Results*) were introduced as predictors of imputation [74]. Ultimately, the total sample of 1240 participants was included in the analyses (311 participants in each experimental condition and 309 participants in each control condition; Figure 1). Analyses were conducted using IBM SPSS Statistics 25.0.

## Results

### Sample

The final sample (N=1240) consisted of physicians (448/1240, 36.13%), nurses (308/1240, 24.84%), dentists (93/1240, 7.50%), physiotherapists (75/1240, 6.05%), midwives (65/1240, 5.24%), paramedics (62/1240, 5.00%), and other medical professionals (191/1240, 15.40%). Overall, job tenure ranged from less than 1 year to 40 years (mean 11.46, SD 10.26), whereas tenure at participants’ current position was mean 6.69 years (SD 7.31). On average, participants worked 47.38 hours per week (SD 18.53). The majority of responders were employed in public health care institutions (924/1240, 74.52%). Women constituted 86.61% (1074/1240) of the sample. Participants’ ages ranged between 20 and 66 years (mean 36.21, SD 10.18). Nearly 17% of the sample had received specialist support (psychotherapy or pharmacotherapy) to cope with job stress at some point in their professional career.

### Preliminary Results

#### Dropout Analysis and Randomization Check

Out of the 1240 participants, 217 completed the posttest assessment and 147 completed the 6-month follow-up. Intervention dropout, defined as a loss to posttest, was 82.50% (1023/1240). The Little [75] test revealed that data were not missing completely at random (*χ*^2^_172_=254.9 *P*<.001). The subsequent ANOVA and chi-square tests confirmed the missing at random (MAR) pattern, depending on the level of primary outcomes at the baseline, condition assignment, and selected job and demographic characteristics. We found that completers were assigned to shorter intervention modules (*χ*^2^_1_=14.9; *P*<.001), were older (*F*_1,1238_=52.10; *P*<.001; η^2^=0.04), with longer total job tenure (*F*_1, 1238_=43.58; *P*<.001; η^2^=0.03) and tenure in the current position (*F*_1,1238_=28.20; *P*<.001; η^2^=0.02), and had been in their respective relationships for longer (*F*_1,1238_=17.11; *P*<.001; η^2^=0.01). They also showed a higher initial expectancy (*F*_1,1238_=12.33; *P*<.001; η^2^=0.01) and perceived credibility of the intervention (*F*_1,1238_=10.57; *P*=.001; η^2^=0.01). Those who completed the intervention presented lower baseline job stress (*F*_1,1238_=9.05; *P*=.003; η^2^=0.01), job burnout (*F*_1,1238_=5.44; *P*=.02; η^2^=0.01), depression (*F*_1,1238_=18.09; *P*<.001; η^2^=0.01), and job-related posttraumatic stress (*F*_1,1238_=7.91; *P*=.005; η^2^=0.01). No other differences were detected. The MAR pattern of missingness justified conducting MI [74].

Participants randomized into 4 study conditions did not differ with regard to either the study variables at baseline or on descriptive characteristics such as age, tenure, and sex. Finally, participants across the 4 conditions did not differ with regard to their expectations of the treatment or its perceived credibility.

Intervention usage was similar between the experimental and control conditions. In the experimental condition reflecting the cultivation process (self-efficacy precedes perceived social support), 209 participants (out of 311 randomized) engaged in at least one exercise in the first module, and 33 of them engaged in at least one exercise in the second module. For the experimental condition reflecting the enabling process (n=311), these numbers were 206 and 43, respectively. In each control condition (n=309), 205 participants engaged in at least one exercise in the self-efficacy or social support enhancement module. Usage of optional modules was similar across the conditions and varied between 98 participants benefiting from at least one optional module in the social support control condition to 124 participants in the self-efficacy control condition.

#### Descriptive Statistics

Means, standard deviations, and Pearson correlations for all measures are found in Multimedia Appendix 1.

#### Effects on Self-Efficacy and Perceived Social Support

We found that there was a significant difference in job stress and burnout-related self-efficacy between time 1 and time 2 (B=−0.08, SE=0.01; 95% CI −0.11 to −0.06) but not between time 2 and time 3 (B=0.00, SE=0.01; 95% CI −0.02 to 0.03). Pairwise comparisons showed that self-efficacy plateaued after posttest but remained significantly higher than at baseline. We observed exactly the same pattern for social support self-efficacy, with a significant difference between time 1 and time 2 (B=−0.18, SE=0.03; 95% CI −0.23 to −0.13) and a nonsignificant difference between time 2 and time 3 (B=0.00, SE=0.02; 95% CI −0.03 to 0.04). In the case of social support, there were significant differences both in the time 1 and time 2 lag (B=−0.19, SE=0.01; 95% CI −0.21 to −0.17) and time 2 and time 3 lag (B=−0.06, SE=0.01; 95% CI −0.08 to −0.03). Pairwise comparisons showed that after social support increased between baseline and posttest, it significantly dropped at the follow-up; however, it remained higher than at baseline.

### Hypotheses Testing

First, for each outcome, we compared a reference model with fixed effects of condition assignment, time, and time by condition interaction as well as 2 random effects associated with each participant—intercept and time—with nested models in which either random effect associated with time or random intercept was not included. For all the outcomes, the differences in –2 Restricted Maximum Likelihood log-likelihood between the reference and nested models were statistically significant, and therefore, we retained both random effects. Moreover, for each of the outcomes, we found them to be statistically significant, indicating that within each study condition, there were considerable variations between participants before they started the intervention and in how they responded over time (Table 2).

**Table 2:** Results of linear mixed effects models.

Outcome		Fixed effects			Random effects	
		Estimate (SE)	95% CI	Estimate (SE)		95% CI
**Job stress**						
	Intercept^a^	2.17-2.23 (0.03)	2.11 to 2.17, 2.22 to 2.45	0.21 (0.01)		0.19-0.23
	SE^b^ to SE+SS^c^	0.02 (0.04)	−0.06 to 0.10	—^d^		—
	SE to SS+SE^e^	−0.03 (0.04)	−0.1 to 0.05	—		—
	SS^f^ to SE+SS	−0.02 (0.04)	−0.10 to 0.06	—		—
	SS to SS+SE	−0.07 (0.04)	−0.15 to 0.01	—		—
	SE+SS to SS+SE	0.05 (0.04)	−0.03 to 0.12	—		—
	Time^a^	−0.08 to −0.09 (0.01)	−0.11 to −0.10, −0.07 to −0.06	0.01 (0.00)		0.01-0.01
	Time×SE to SE+SS	−0.05 (0.01)	−0.08 to −0.03	—		—
	Time×SE to SS+SE	0.01 (0.01)	−0.01 to 0.04	—		—
	Time×SS to SE+SS	−0.07 (0.01)	−0.09 to−0.04	—		—
	Time×SS to SS+SE	0.00 (0.01)	−0.03 to 0.03	—		—
	Time×SE+SS to SS+SE	−0.07 (0.01)	−0.09 to −0.04	—		—
**Job burnout**						
	Intercept^a^	2.61-2.70 (0.03)	2.56 to 2.65, 2.67 to 2.76	0.21 (0.01)		0.19-0.24
	SE to SE+SS	0.03 (0.04)	−0.05 to 0.11	—		—
	SE to SS+SE	0.04 (0.04)	−0.03 to 0.12	—		—
	SS to SE+SS	−0.06 (0.04)	−0.14 to 0.02	—		—
	SS to SS+SE	−0.05 (0.04)	−0.12 to 0.03	—		—
	SE+SS to SS+SE	−0.01 (0.04)	−0.09 to 0.06	—		—
	Time^a^	−0.02 to −0.06 (0.01)	−0.08 to −0.03, −0.05 to −0.00	0.01 (0.00)		0.00-0.01
	Time×SE to SE+SS	−0.03 (0.01)	−0.05 to −0.00	—		—
	Time×SE to SS+SE	−0.04 (0.01)	−0.04 to −0.00	—		—
	Time×SS to SE+SS	0.02 (0.01)	−0.00 to 0.05	—		—
	Time×SS to SS+SE	0.01 (0.01)	−0.02 to 0.03	—		—
	Time×SE+SS to SS+SE	0.02 (0.01)	−0.01 to 0.04	—		—
**Work engagement**						
	Intercept^a^	4.06-4.21 (0.06)	3.95 to 4.09, 4.18 to 4.32	0.89 (0.04)		0.81-0.98
	SE to SE+SS	−0.03 (0.08)	−0.19 to 0.13	—		—
	SE to SS+SE	−0.04 (0.08)	−0.20 to 0.12	—		—
	SS to SE+SS	0.12 (0.08)	−0.04 to 0.28	—		—
	SS to SS+SE	0.10 (0.08)	−0.06 to 0.26	—		—
	SE+SS to SS+SE	0.01 (0.08)	0.15 to 0.17	—		—
	Time^a^	−0.09 to −0.19 (0.02)	−0.23 to −0.13, −0.16 to −0.05	0.03 (0.01)		0.02-0.04
	Time×SE to SE+SS	−0.05 (0.03)	−0.10 to 0.01	—		—
	Time×SE to SS+SE	−0.02 (0.03)	−0.07 to 0.04	—		—
	Time×SS to SE+SS	−0.14 (0.03)	−0.19 to −0.08	—		—
	Time×SS to SS+SE	−0.10 (0.03)	−0.16 to −0.05	—		—
	Time×SE+SS to SS+SE	−0.03 (0.03)	−0.09 to 0.02	—		—
**Depression**						
	Intercept^a^	1.16-1.21 (0.04)	1.08 to 1.13, 1.23 to 1.28	0.36 (0.02)		0.33, 0.39
	SE to SE+SS	0.05 (0.05)	−0.06 to 0.15	—		—
	SE to SS+SE	−0.05 (0.05)	−0.15 to 0.05	—		—
	SS to SE+SS	0.05 (0.05)	−0.05 to 0.15	—		—
	SS to SS+SE	−0.05 (0.05)	−0.15 to 0.06	—		—
	SE+SS to SS+SE	0.09 (0.05)	−0.01 to 0.20	—		—
	Time	−0.04 to −0.07 (0.01)	−0.10 to −0.06, −0.05 to −0.01	0.02 (0.00)		0.02, 0.02
	Time×SE to SE+SS	−0.01 (0.01)	−0.05 to 0.02	—		—
	Time×SE to SS+SE	0.01 (0.02)	−0.02 to 0.05	—		—
	Time×SS to SE+SS	−0.05 (0.02)	−0.08 to −0.01	—		—
	Time×SS to SS+SE	−0.02 (0.02)	−0.06 to 0.01	—		—
	Time×SE+SS to SS+SE	−0.03 (0.02)	−0.06 to 0.01	—		—
**Job-related traumatic stress^g^**						
	Intercept^a^	1.26-1.38 (0.05)	1.17 to 1.29, 1.36 to 1.48	0.60 (0.03)		0.54, 0.65
	SE to SE+SS	−0.10 (0.07)	−0.23 to 0.03	—		—
	SE to SS+SE	−0.09 (0.07)	−0.22 to 0.04	—		—
	SS to SE+SS	0.02 (0.07)	−0.11 to 0.16	—		—
	SS to SS+SE	0.03 (0.07)	−0.10 to 0.16	—		—
	SE+SS to SS+SE	−0.01 (0.07)	−0.14 to 0.12	—		—
	Time^a^	0.00-0.03 (0.02)	−0.06 to −0.00, 0.01 to 0.07	0.05 (0.00)		0.04, 0.06
	Time×SE to SE+SS	−0.02 (0.03)	−0.07 to 0.03	—		—
	Time×SE to SS+SE	0.03 (0.03)	−0.02 to 0.08	—		—
	Time×SS to SE+SS	−0.07 (0.03)	−0.12 to −0.02	—		—
	Time×SS to SS+SE	−0.03 (0.03)	−0.08 to 0.02	—		—
	Time×SE+SS to SS+SE	−0.04 (0.03)	−0.09 to 0.01	—		—

^a^As the values differ slightly depending on which study condition is used as a reference category, a range of estimates and confidence intervals are provided.

^b^SE: self-efficacy enhancement module.

^c^SE+SS: self-efficacy and perceived social support sequential enhancement module.

^d^The lack of data in these cells follows statistical analysis: these values are only provided for intercept and time.

^e^SS+SE: perceived social support and self-efficacy sequential enhancement module.

^f^SS: perceived social support enhancement module.

^g^Controlling for exposure to traumatic events.

We expected that the experimental conditions (ie, the ones comprised 2 modules: SE+SS and SS+SE) would be more effective than each active control condition (ie, SE and SS) in reducing job stress and job burnout (primary outcomes) as well as depression and job-related posttraumatic stress (secondary outcomes) at posttest (time 2), and we expected these effects to remain at a 6-month follow-up (time 3). In the case of work engagement, we expected it to be higher at time 2 in the experimental conditions in comparison with active controls, and we expected the effects to remain at a 6-month follow-up (time 3). We were also interested in whether the 2 experimental conditions would differ in their efficacy.

### Job Stress

The mixed effects model for job stress showed no significant effect of condition assignment on job stress for any of the between-group comparisons, but it did show a significant effect of time for all comparisons (Table 2), indicating that stress decreased with time. Significant interaction effects were found for time and the comparison between the experimental condition SE+SS with active control SE (B=−0.05, SE=0.01; (95% CI −0.08 to −0.03) and with active SS control (B=−0.06, SE=0.01; 95% CI −0.09 to −0.04) as well as for time and the comparison between the 2 experimental groups (B=−0.07, SE=0.01; 95% CI −0.09 to −0.04). Bonferroni post hoc tests revealed that at time 2, job stress was significantly lower in the experimental SE+SS condition when compared with the active control SS (*d*=−0.21; 95% CI −0.37 to −0.05). However, at time 3, it was lower in the SE+SS condition than in the remaining 3 conditions: for SS, *d*=−0.41, 95% CI −0.57 to −0.25; for SE, *d*=−0.24, 95% CI −0.40 to −0.09; and for SS+SE, *d*=−0.24, 95% CI −0.39 to −0.08.

### Job Burnout

Assignment had no main effect on job burnout for either of the comparisons between study conditions, but there was a main effect of time for all the comparisons (Table 2), indicating that burnout decreased with time. Interaction effects of time and the comparisons between groups were either not significant or marginally significant at a level that did not warrant further investigation due to the risk of inflated type I error resulting from multiple comparisons.

### Work Engagement

We found no main effect of condition assignment on work engagement, but we did find a main effect of time (Table 2) for the comparisons between all study conditions, showing that work engagement decreased with time. Significant interactions were found for time and the comparisons between SS active control and 2 experimental groups: SE+SS (B=−0.13, SE=0.03; 95% CI −0.19 to −0.08) and SS+SE (B=−0.10, SE=0.03; 95% CI −0.16 to −0.05). Bonferroni post hoc tests demonstrated that there were no significant differences between study conditions at time 2, but at time 3, work engagement was significantly lower in the experimental condition SE+SS than in the active control SS (*d*=−0.20; 95% CI −0.36 to −0.04).

### Depression

Similar to the case of work engagement, for depression, we found an effect of time, indicating that depression decreased with time, but no effect of condition assignment for any of the between-group comparisons (Table 2). The only significant interaction was found for time and the comparison between active control SS and experimental condition SE+SS (B=−0.05, SE=0.02; 95% CI −0.08 to −0.01). However, Bonferroni post hoc tests revealed that there were no meaningful differences between these conditions at either posttest or at follow-up.

### Job-Related Traumatic Stress

For job-related traumatic stress, we additionally controlled for the fixed effect of the exposure to traumatic events. Neither condition assignment nor time had an effect on the outcome (Table 2). A significant interaction was observed for time and the comparison between active control SS and experimental SE+SS condition (B=−0.07, SE=0.03; 95% CI −0.12 to −0.02). Bonferroni post hoc analysis showed that this difference was significant only at time 3, with lower stress in the experimental condition (*d*=−0.24; 95% CI −0.40 to −0.08).

### Predictors of Outcomes

To identify potential predictors of the 2 primary outcomes, job stress and job burnout, we ran bootstrapped regression analyses with the outcomes reflecting change between pretest and posttest (residual change score) and 95% bias-corrected bootstrap CIs. Age, gender, marital status, average weekly work hours, type of organization (public vs private), receiving treatment in the past, and general tenure were not associated with changes in job stress and job burnout. However, tenure in the current workplace turned out to be a significant predictor of both change in job stress (β=−.22; 95% CI −0.02 to −0.01) and in burnout (β=−.18; 95% CI −0.02 to −0.01): greater reduction in both outcomes was observed among participants with shorter tenure. Changes in job stress and burnout were also associated with the level of expectations of the intervention. Participants who had higher expectations of the treatment and perceived the intervention as more credible experienced a greater decrease in stress (credibility: β=.16; 95% CI 0.04-0.09 and expectancy: β=.17; 95% CI 0.04-0.09) and in burnout (credibility: β=.10; 95% CI 0.01-0.06 and expectancy: β=.25; 95% CI 0.06-0.10).

In terms of using the intervention, change in job stress was predicted by engaging in exercises in the self-efficacy enhancing module (β=.11; 95% CI 0.06-0.22): at the posttest, stress was lower among the participants who engaged in the self-efficacy module. Using social support enhancing exercises and optional modules (relaxation, mindfulness, cognitive restructuring, and lifestyle) was not associated with change in job stress. A change in job burnout was predicted neither by engagement in self-efficacy nor by social support exercises. Only using optional modules was associated with a reduction in job burnout at the posttest (β=.07; 95% CI 0.01-0.16).

## Discussion

### Principal Findings

This study had 2 objectives. First, we wanted to test whether enhancing 2 psychological resources, perceived social support and self-efficacy, would be more effective for improving the well-being of medical professionals than strengthening only 1 of them. Second, we aimed to experimentally verify the enabling versus the cultivation hypothesis, that is, to test whether the sequence in which social support and self-efficacy were targeted would have an effect on well-being. To reflect the complexity of a natural occupational context, we tested the efficacy of the intervention for 5 outcomes. We found that job burnout did not depend on assignment to the study condition. However, allocation to study condition did have an effect on another primary outcome, job stress. Immediately after the intervention, we found that one experimental condition (ie, the one reflecting the cultivation hypothesis by first enhancing self-efficacy and later social support) was more effective in reducing stress than the control condition that solely targeted social support, but not the one dedicated to enhancing only self-efficacy. This result partially supported our expectations. However, 6 months later, the same experimental condition was found to be the most effective. This result supported the cultivation process of stress reduction over an enabling one. When we analyzed how participants used the Med-Stress intervention regardless of condition assignment, we found that there was a reduction in job burnout, and it was predicted by using optional modules that comprised CBT-based exercises. Perhaps these exercises, and not those dedicated to building personal resources (ie, self-efficacy and perceived social support), were responsible for the decrease in burnout, albeit small, in participants across all conditions. Job stress, on the other hand, was not associated with completing optional modules; the only significant predictor of its decrease was using self-efficacy exercises.

No meaningful differences between study conditions were detected for depression. In the case of job-related traumatic stress, between-group differences at the posttest were negligible, whereas at the follow-up, we again found that participants in the condition reflecting the cultivation hypothesis reported greater reduction in stress than those in a control condition enhancing solely social support, although the effect was small. Work engagement also revealed different patterns at posttest and follow-up. Immediately after the intervention, there were no meaningful differences in work engagement between study conditions; however, 6 months later, work engagement was lower in the same experimental condition reflecting the cultivation hypothesis than in the control condition that aimed to build only social support. Moreover, contrary to what might be expected, work engagement decreased over time. In fact, at the follow-up, work engagement was significantly lower in the same condition that we found was beneficial for the reduction of both types of job-related stress. In other words, participants who first completed self-efficacy enhancing exercises and then the ones dedicated to building perception of social support reported lower stress and lower work engagement. This pattern of results suggests that a decrease in work engagement might be beneficial to medical professionals. In fact, although work engagement is overly considered a positive state, associated with numerous beneficial consequences for a person, such as better self-reported health, a so-called dark side of engagement has also been identified, suggesting that over-engaged workers might experience undesired outcomes [76]. Moreover, the results of a recent meta-analysis [77] showed that only half of the interventions aimed at improving work engagement were found to have a positive effect. The rest had no effect or had a negative effect, although the latter were in the minority. Interestingly, the intervention that resulted in decreased work engagement was dedicated to service workers in older care, a group that is similar to the one in Med-Stress [78]. This might indicate that, at least for these occupational groups, being highly engaged in work can be detrimental. At the same time, this interpretation needs to be treated with caution as one of the options that require further analysis.

Taken together, these results show that the experimental condition reflecting the cultivation hypothesis—self-efficacy preceding social support—is the most beneficial in the long run, although this finding needs to be treated with caution for the following reasons: first, it was not found for all components of well-being. Second, for those that it was found (job stress and traumatic stress), the effect sizes were small. Third, in the case of work engagement, more research is needed to establish whether the decrease over time was beneficial or detrimental to the participants. Small effect sizes are perhaps not that surprising considering that gain cycles, contrary to loss spirals, are considered to be weaker and take longer to unfold [23]. As 6 months is a relatively short period, subsequent measurement points are needed, and therefore, we have scheduled another follow-up in the future. In addition, small effect sizes could be attributed to the comparisons being made between experimental conditions and active, not passive, controls [79].

Perhaps reaching a result that is not completely coherent for all outcomes is not that surprising when these outcomes are numerous and represent different aspects of well-being. By targeting multiple outcomes with one program, we risk losing precision, but we gain a more naturalistic intervention.

### Strengths and Limitations

Med-Stress responds to a call “to conduct feasibility studies and randomized controlled studies on the effect of low-intensity interventions and technology supported (eg, web-based) interventions in low- and middle-income countries (LMICs), preferably using an active control condition as comparison, to ensure we disseminate effective treatments in LMICs” [80]. LMICs are reported to experience a disparity between the need for mental health services and their provision. We would argue that the need for such treatments is also urgent among certain groups, including health care providers. Content development of Med-Stress was preceded by a web-based focus study to better understand the working conditions of the target audience. We found that despite high levels of stress and burnout among medical professionals, no support programs were offered at the organizations where responders worked. Benefiting from traditional help often proves difficult for them due to irregular working schedules and an insufficient number of professionals. Thus, the availability of an internet intervention of low intensity and focused on personal resources that are context-free could be beneficial in alleviating some work-related mental health issues. This became particularly salient a few months later, when the COVID-19 pandemic broke and medical professionals found themselves in need of support under conditions where it could not be easily provided. In fact, to respond to this crisis, we developed a shorter, contextualized version of the Med-Stress intervention, called Med-Stress SOS [81].

Our study has several limitations. First, the dropout was high, which affects the interpretation of the results. We mitigated this risk by applying the MI method. There is an ongoing argument regarding the threshold over which the dropout rate is deemed unacceptable; however, the latest simulations indicate that it is not the proportion but the pattern of missingness that should be taken into account when considering data imputation. Specifically, in the case of the MAR pattern, the MI procedure, which includes all identified variables that differentiate dropouts from completers as imputation predictors, leads to the least biased results [74,82]. As the pattern of missingness in this study was indeed MAR, we followed these recommendations when conducting MI. Moreover, it should be said that internet interventions, in particular self-guided ones, do tend to have high dropout rates. Although such a loss to follow-up is rare, it is not unprecedented [83,84]. However, despite our attempts to mitigate the risks resulting from high dropout, the obtained results need to be treated with caution. This loss reflects the pragmatic nature of this trial: it was conducted in Poland, where internet interventions are still rare, and we suspect that users did not have a framework into which this form of psychological help could be easily incorporated. A high recruitment rate compared with high dropout probably reflects participants’ initial enthusiasm and curiosity that diminished over time. This was also reflected in the poor usage of the intervention, particularly in the experimental conditions in which the second assigned module was used much less frequently than the first one. In fact, comments that we received post intervention seem to support this notion: users referred to the content of the exercises (not user-friendly enough), pace of the intervention (new exercise released each week), and the fact that it was a web-based intervention and not an app-based intervention. High dropout was also a reason why we could not conduct a per protocol analysis: the sample of completers was too small to provide sufficient power. Ultimately, we had enough power to detect effects of minimum *d*=0.21, and therefore, all findings could not be generalized; however, the effects of job stress and job-related traumatic stress were large enough. Some of the phenomena in this study, in particular job burnout and work engagement, are multidimensional, and analyzing them as such could provide a more in-depth understanding of the intervention’s impact on them. However, this would further increase the already high number of comparisons that needed to be accounted for in the study design.

### Conclusions

This study offers both theoretical and practical contributions. It is a pragmatic trial that offers an insight into how people really use this intervention [85]. It is an experimental verification of the cultivation versus enabling process of stress reduction, demonstrating cautious support for the former. Moreover, we empirically tested the concept of gain cycles for 5 components of well-being. In practical terms, Med-Stress is a support program that was found to be effective in enhancing the well-being of medical professionals. This intervention has the advantage of being broadly accessible to health care workers who currently receive no help in coping with their mental health problems.

## Appendix

Multimedia Appendix 1 Means, SDs, and correlations for study variables.

Multimedia Appendix 2 CONSORT-EHEALTH checklist (V 1.6.1).

## Abbreviations

ANOVA: analysis of variance
CBT: cognitive behavioral therapy
COR: conservation of resources theory
LMIC: low- and middle-income country
MAR: missing at random
MI: multiple imputation
RCT: randomized controlled trial
SE: self-efficacy enhancement module
SE+SS: self-efficacy and perceived social support sequential enhancement module
SS: perceived social support enhancement module
SS+SE: perceived social support and self-efficacy sequential enhancement module

## References

[1] Wright TA, Cropanzano R (2000). Psychological well-being and job satisfaction as predictors of job performance. J Occup Health Psychol.

[2] Darr W, Johns G (2008). Work strain, health, and absenteeism: a meta-analysis. J Occup Health Psychol.

[3] Fried Y, Shirom A, Gilboa S, Cooper CL (2008). The mediating effects of job satisfaction and propensity to leave on role stress-job performance relationships: Combining meta-analysis and structural equation modeling. Int J Stress Manag.

[4] Brand SL, Coon JT, Fleming LE, Carroll L, Bethel A, Wyatt K (2017). Whole-system approaches to improving the health and wellbeing of healthcare workers: a systematic review. PLoS One.

[5] Rothenberger DA (2017). Physician burnout and well-being: a systematic review and framework for action. Dis Colon Rectum.

[6] West CP, Dyrbye LN, Erwin PJ, Shanafelt TD (2016). Interventions to prevent and reduce physician burnout: a systematic review and meta-analysis. Lancet.

[7] Rickard G, Lenthall S, Dollard M, Opie T, Knight S, Dunn S, Wakerman J, MacLeod M, Seller Jo, Brewster-Webb D (2012). Organisational intervention to reduce occupational stress and turnover in hospital nurses in the Northern territory, Australia. Collegian.

[8] Nielsen K, Nielsen MB, Ogbonnaya C, Känsälä M, Saari E, Isaksson K (2017). Workplace resources to improve both employee well-being and performance: a systematic review and meta-analysis. Work & Stress.

[9] Joyce S, Modini M, Christensen H, Mykletun A, Bryant R, Mitchell P B, Harvey S B (2016). Workplace interventions for common mental disorders: a systematic meta-review. Psychol Med.

[10] Lomas T, Medina JC, Ivtzan I, Rupprecht S, Eiroa-Orosa FJ (2018). Mindfulness-based interventions in the workplace: an inclusive systematic review and meta-analysis of their impact upon wellbeing. J Posit Psychol.

[11] Andersson G, Carlbring P, Titov N, Lindefors N (2019). Internet interventions for adults with anxiety and mood disorders: a narrative umbrella review of recent meta-analyses. Can J Psychiatry.

[12] Carl E, Stein AT, Levihn-Coon A, Pogue JR, Rothbaum B, Emmelkamp P, Asmundson GJ, Carlbring P, Powers MB (2019). Virtual reality exposure therapy for anxiety and related disorders: a meta-analysis of randomized controlled trials. J Anxiety Disord.

[13] Carlbring P, Andersson G, Cuijpers P, Riper H, Hedman-Lagerlöf E (2018). Internet-based vs. face-to-face cognitive behavior therapy for psychiatric and somatic disorders: an updated systematic review and meta-analysis. Cogn Behav Ther.

[14] Carolan S, Harris PR, Cavanagh K (2017). Improving employee well-being and effectiveness: systematic review and meta-analysis of web-based psychological interventions delivered in the workplace. J Med Internet Res.

[15] Stratton E, Lampit A, Choi I, Calvo RA, Harvey SB, Glozier N (2017). Effectiveness of eHealth interventions for reducing mental health conditions in employees: a systematic review and meta-analysis. PLoS One.

[16] Geraedts AS, Kleiboer AM, Twisk J, Wiezer NM, van Mechelen W, Cuijpers P (2014). Long-term results of a web-based guided self-help intervention for employees with depressive symptoms: randomized controlled trial. J Med Internet Res.

[17] Aikens KA, Astin J, Pelletier KR, Levanovich K, Baase CM, Park YY, Bodnar CM (2014). Mindfulness goes to work: impact of an online workplace intervention. J Occup Environ Med.

[18] Ebert DD, Heber E, Berking M, Riper H, Cuijpers P, Funk B, Lehr D (2016). Self-guided internet-based and mobile-based stress management for employees: results of a randomised controlled trial. Occup Environ Med.

[19] Heber E, Lehr D, Ebert DD, Berking M, Riper H (2016). Web-based and mobile stress management intervention for employees: a randomized controlled trial. J Med Internet Res.

[20] Lazarus R, Folkman S (1984). Stress, Appraisal, and Coping.

[21] Demerouti E, Nachreiner F, Bakker AB, Schaufeli WB (2001). The Job Demands-Resources Model of Burnout. Journal of Applied Psychology.

[22] Hobfoll SE (2001). The influence of culture, community, and the nested‐self in the stress process: advancing conservation of resources theory. Appl Psychol.

[23] Hobfoll SE, Halbesleben J, Neveu J, Westman M (2018). Conservation of resources in the organizational context: the reality of resources and their consequences. Annu Rev Organ Psychol Organ Behav.

[24] Shoji K, Lesnierowska M, Smoktunowicz E, Bock J, Luszczynska A, Benight CC, Cieslak R (2015). What comes first, job burnout or secondary traumatic stress? Findings from two longitudinal studies from the U.S. And Poland. PLoS One.

[25] Llorens S, Schaufeli W, Bakker A, Salanova M (2007). Does a positive gain spiral of resources, efficacy beliefs and engagement exist?. Comput Hum Behav.

[26] Salanova M, Schaufeli WB, Xanthopoulou D, Bakker AB, Bakker AB, Leiter MP (2010). The gain spiral of resources and work engagement: Sustaining a positive worklife. Work Engagement: A Handbook of Essential Theory and Research.

[27] Seppälä P, Hakanen J, Mauno S, Perhoniemi R, Tolvanen A, Schaufeli W (2014). Stability and change model of job resources and work engagement: a seven-year three-wave follow-up study. Eur J Work Organ Psychol.

[28] Kim SD, Hollensbe EC, Schwoerer CE, Halbesleben JRB (2015). Dynamics of a wellness program: a conservation of resources perspective. J Occup Health Psychol.

[29] Nurullah A (2012). Received and provided social support: a review of current evidence and future directions. Am J Health Stud.

[30] Sarason BR, Sarason IG, Pierce GR (1990). Traditional views of social support and their impact on assessment. Social Support: An Interactional View. Wiley series on personality processes.

[31] Haber MG, Cohen JL, Lucas T, Baltes BB (2007). The relationship between self-reported received and perceived social support: a meta-analytic review. Am J Community Psychol.

[32] Hobfoll SE, Freedy J, Lane C, Geller P (2016). Conservation of social resources: social support resource theory. J Soc Pers Relatsh.

[33] Bandura A (1997). Self-Efficacy: The Exercise of Control.

[34] Byars-Winston A, Diestelmann J, Savoy JN, Hoyt WT (2017). Unique effects and moderators of effects of sources on self-efficacy: a model-based meta-analysis. J Couns Psychol.

[35] Bandura A (1986). Social Foundations of Thought and Action: A Social Cognitive Theory.

[36] Schwarzer R, Knoll N (2007). Functional roles of social support within the stress and coping process: a theoretical and empirical overview. Int J Psychol.

[37] Hohl DH, Schultze M, Keller J, Heuse S, Luszczynska A, Knoll N (2019). Inter-relations between partner-provided support and self-efficacy: a dyadic longitudinal analysis. Appl Psychol Health Well Being.

[38] Benight CC, Bandura A (2004). Social cognitive theory of posttraumatic recovery: the role of perceived self-efficacy. Behav Res Ther.

[39] Shoji K, Bock J, Cieslak R, Zukowska K, Luszczynska A, Benight CC (2014). Cultivating secondary traumatic growth among healthcare workers: the role of social support and self-efficacy. J Clin Psychol.

[40] Parent-Thirion A, Biletta I, Cabrita J, Aleksandra W, Oscar V (2017). 6th European Working Conditions Survey: Overview Report 2017 update.

[41] Wallace JE, Lemaire JB, Ghali WA (2009). Physician wellness: a missing quality indicator. Lancet.

[42] Cohen S, Kamarck T, Mermelstein R (1983). A global measure of perceived stress. J Health Soc Behav.

[43] (2018). International Classification of Diseases for Mortality and Morbidity Statistics (11th Revision). World Health Organization.

[44] Maslach C, Schaufeli WB, Leiter MP (2001). Job burnout. Annu Rev Psychol.

[45] Schaufeli WB, Leiter MP, Maslach C (2009). Burnout: 35 years of research and practice. Career Dev Int.

[46] Zgliczyńska M, Zgliczyński S, Ciebiera M, Kosińska-Kaczyńska K (2019). Occupational burnout syndrome in Polish physicians: a systematic review. Int J Environ Res Public Health.

[47] Dubale BW, Friedman LE, Chemali Z, Denninger JW, Mehta DH, Alem A, Fricchione GL, Dossett ML, Gelaye B (2019). Systematic review of burnout among healthcare providers in sub-Saharan Africa. BMC Public Health.

[48] Imai H, Nakao H, Nakagi Y, Niwata S, Sugioka Y, Itoh T, Yoshida T (2006). Prevalence of burnout among public health nurses in charge of mental health services and emergency care systems in Japan. Environ Health Prev Med.

[49] Rotenstein LS, Torre M, Ramos MA, Rosales RC, Guille C, Sen S, Mata DA (2018). Prevalence of burnout among physicians: a systematic review. J Am Med Assoc.

[50] Schaufeli W, Salanova M, González-romá V, Bakker A (2002). The measurement of engagement and burnout: a two sample confirmatory factor analytic approach. J Happiness Stud.

[51] Moeller J, Ivcevic Z, White A, Menges J, Brackett M (2018). Highly engaged but burned out: intra-individual profiles in the US workforce. Career Dev Int.

[52] American Psychiatric Association (2013). Diagnostic and statistical manual of mental disorders DSM-5.

[53] Beck CT (2011). Secondary traumatic stress in nurses: a systematic review. Arch Psychiatr Nurs.

[54] Nimmo A, Huggard P (2013). A systematic review of the measurement of compassion fatigue, vicarious trauma, and secondary traumatic stress in physicians. Australas J Disaster Trauma Stud.

[55] Harvey SB, Glozier N, Henderson M, Allaway S, Litchfield P, Holland-Elliott K, Hotopf M (2011). Depression and work performance: an ecological study using web-based screening. Occup Med (Lond).

[56] Beiwinkel T, Eißing T, Telle N, Siegmund-Schultze E, Rössler W (2017). Effectiveness of a web-based intervention in reducing depression and sickness absence: randomized controlled trial. J Med Internet Res.

[57] van Dam A (2016). Subgroup analysis in burnout: relations between fatigue, anxiety, and depression. Front Psychol.

[58] Smoktunowicz E, Lesnierowska M, Cieslak R, Carlbring P, Andersson G (2019). Efficacy of an Internet-based intervention for job stress and burnout among medical professionals: study protocol for a randomized controlled trial. Trials.

[59] Faul F, Erdfelder E, Buchner A, Lang A (2009). Statistical power analyses using G*Power 3.1: tests for correlation and regression analyses. Behav Res Methods.

[60] Heber E, Ebert DD, Lehr D, Cuijpers P, Berking M, Nobis S, Riper H (2017). The benefit of web- and computer-based interventions for stress: a systematic review and meta-analysis. J Med Internet Res.

[61] Magnusson, K (2018). Power Analysis for Longitudinal 2- and 3-Level Models Challenges and Some Solutions Using the R Package powerlmm. R Psychologist.

[62] Vlaescu G, Alasjö A, Miloff A, Carlbring P, Andersson G (2016). Features and functionality of the Iterapi platform for internet-based psychological treatment. Internet Interv.

[63] Demerouti E, Mostert K, Bakker AB (2010). Burnout and work engagement: a thorough investigation of the independency of both constructs. J Occup Health Psychol.

[64] Kroenke K, Spitzer RL, Williams JB (2001). The PHQ-9: validity of a brief depression severity measure. J Gen Intern Med.

[65] Schaufeli WB, Shimazu A, Hakanen J, Salanova M, De Witte H (2019). An ultra-short measure for work engagement. Eur J Psychol Assess.

[66] Weathers F, Litz B, Keane T, Palmieri P, Marx B, Schnurr P (2013). PTSD Checklist for DSM-5 (PCL-5). PTSD: National Center for PTSD.

[67] Lua H (2008). The Mediating Role of Work Stress and Burnout Management Self-efficacy in the Job Demand - Resources Model [dissertation].

[68] Schwarzer R, Schulz U (2000). Berlin Social Support Scales (BSSS). Measurement Instrument Database for the Social Sciences.

[69] Widerszal-Bazyl M, Cieślak R (2000). Monitoring psychosocial stress at work: development of the psychosocial working conditions questionnaire. Int J Occup Saf Ergon.

[70] Cieslak R, Shoji K, Luszczynska A, Taylor S, Rogala A, Benight CC (2013). Secondary trauma self-efficacy: concept and its measurement. Psychol Assess.

[71] Devilly GJ, Borkovec TD (2000). Psychometric properties of the credibility/expectancy questionnaire. J Behav Ther Exp Psychiatry.

[72] West B, Welch K, Galecki A (2015). Linear Mixed Models. A practical guide using statistical software, Second Edition.

[73] Steketee G, Chambless D (1992). Methodological issues in prediction of treatment outcome. Clin Psychol Rev.

[74] Groenwold RH, Donders AR, Roes KC, Harrell FE, Moons KG (2012). Dealing with missing outcome data in randomized trials and observational studies. Am J Epidemiol.

[75] Little R, Rubin D (2002). Statistical Analysis with Missing Data (2 edition).

[76] Bakker AB, Albrecht SL, Leiter MP (2011). Key questions regarding work engagement. Eur J Work Organ Psychol.

[77] Knight C, Patterson M, Dawson J (2019). Work engagement interventions can be effective: a systematic review. Eur J Work Organ Psychol.

[78] Ng S (2014). Is brief daily body–mind–spirit practice desirable for staff who provide services for elderly people? Two pilot studies with care and professional workers. Asia Pac J Soc Work Dev.

[79] Emanuel EJ, Miller FG (2001). The ethics of placebo-controlled trials--a middle ground. N Engl J Med.

[80] Bockting CL, Williams AD, Carswell K, Grech AE (2016). The potential of low-intensity and online interventions for depression in low- and middle-income countries. Glob Ment Health (Camb).

[81] Smoktunowicz E, Lesnierowska M, Cieslak R, Carlbring P, Andersson G (2020). Med-Stress SOS: Internet Intervention for Medical Professionals Working During Health Crisis. Center for Open Science.

[82] Madley-Dowd P, Hughes R, Tilling K, Heron J (2019). The proportion of missing data should not be used to guide decisions on multiple imputation. J Clin Epidemiol.

[83] Eysenbach Gunther (2005). The law of attrition. J Med Internet Res.

[84] Richards D, Richardson T (2012). Computer-based psychological treatments for depression: a systematic review and meta-analysis. Clin Psychol Rev.

[85] Tosh G, Soares-Weiser K, Adams CE (2011). Pragmatic vs explanatory trials: the pragmascope tool to help measure differences in protocols of mental health randomized controlled trials. Dialogues Clin Neurosci.

