# Supplementary material for: Resource-Based Internet Intervention (Med-Stress) to Improve Well-Being Among Medical Professionals: Randomized Controlled Trial
Source: J Med Internet Res. 2021 Jan 11;23(1):e21445. doi: 10.2196/21445 (PMC7834939; doi:10.2196/21445)
Supplement: Multimedia Appendix 1 [file jmir_v23i1e21445_app1.docx]

Appendix

*Means, Standard Deviations, and Correlations for Study Variables*

| **Variable** | ***M*** | ***SD*** | **Range** | **1** | **2** | **3** | **4** | **5** | **6** | **7** | **8** | **9** | **10** | **11** | **12** | **13** | **14** | **15** | **16** | **17** | **18** | **19** | **20** | **21** | **22** | **23** | **24** | **25** | **26** | **27** |
| --- | --- | --- | --- | --- | --- | --- | --- | --- | --- | --- | --- | --- | --- | --- | --- | --- | --- | --- | --- | --- | --- | --- | --- | --- | --- | --- | --- | --- | --- | --- |
| 1. Job Stress T1 | 2.23 | 0.52 | 0—4 | – |  |  |  |  |  |  |  |  |  |  |  |  |  |  |  |  |  |  |  |  |  |  |  |  |  |  |
| 2. Job Stress T2 | 2.04 | 0.38 | 0—4 | .79^**^ | – |  |  |  |  |  |  |  |  |  |  |  |  |  |  |  |  |  |  |  |  |  |  |  |  |  |
| 3. Job Stress T3 | 2.03 | 0.39 | 0—4 | .73^**^ | .78^**^ | – |  |  |  |  |  |  |  |  |  |  |  |  |  |  |  |  |  |  |  |  |  |  |  |  |
| 4. Job Burnout T1 | 2.68 | 0.52 | 1—4 | .62^**^ | .61^**^ | .55^**^ | – |  |  |  |  |  |  |  |  |  |  |  |  |  |  |  |  |  |  |  |  |  |  |  |
| 5. Job Burnout T2 | 2.56 | 0.40 | 1—4 | .61^**^ | .78^**^ | .60^**^ | .84^**^ | – |  |  |  |  |  |  |  |  |  |  |  |  |  |  |  |  |  |  |  |  |  |  |
| 6. Job Burnout T3 | 2.59 | 0.39 | 1—4 | .54^**^ | .64^**^ | .70^**^ | .84^**^ | .80^**^ | – |  |  |  |  |  |  |  |  |  |  |  |  |  |  |  |  |  |  |  |  |  |
| 7. Work Engagement T1 | 4.19 | 1.03 | 0—6 | -.27^**^ | -.33^**^ | -.28^**^ | -.54^**^ | -.50^**^ | -.50^**^ | – |  |  |  |  |  |  |  |  |  |  |  |  |  |  |  |  |  |  |  |  |
| 8. Work Engagement T1 | 3.91 | 0.87 | 0—6 | -.32^**^ | -.43^**^ | -.34^**^ | -.55^**^ | -.60^**^ | -.59^**^ | .83^**^ | – |  |  |  |  |  |  |  |  |  |  |  |  |  |  |  |  |  |  |  |
| 9. Work Engagement T1 | 3.84 | 0.79 | 0—6 | -.30^**^ | -.37^**^ | -.39^**^ | -.62^**^ | -.57^**^ | -.64^**^ | .75^**^ | .71^**^ | – |  |  |  |  |  |  |  |  |  |  |  |  |  |  |  |  |  |  |
| 10. Depression T1 | 1.24 | 0.69 | 0—3 | .67^**^ | .58^**^ | .63^**^ | .63^**^ | .60^**^ | .54^**^ | -.26^**^ | -.24^**^ | -.33^**^ | – |  |  |  |  |  |  |  |  |  |  |  |  |  |  |  |  |  |
| 11. Depression T2 | 1.07 | 0.45 | 0—3 | .56^**^ | .62^**^ | .63^**^ | .52^**^ | .63^**^ | .53^**^ | -.13^**^ | -.25^**^ | -.25^**^ | .82^**^ | – |  |  |  |  |  |  |  |  |  |  |  |  |  |  |  |  |
| 12. Depression T3 | 1.11 | 0.42 | 0—3 | .51^**^ | .55^**^ | .72^**^ | .56^**^ | .57^**^ | .66^**^ | -.25^**^ | -.27^**^ | -.41^**^ | .79^**^ | .79^**^ | – |  |  |  |  |  |  |  |  |  |  |  |  |  |  |  |
| 13. Job-Related Traumatic Stress T1 | 1.37 | 0.88 | 0—4 | .50^**^ | .39^**^ | .40^**^ | .42^**^ | .45^**^ | .30^**^ | -.13^**^ | -.13^**^ | -.16^**^ | .63^**^ | .52^**^ | .45^**^ | – |  |  |  |  |  |  |  |  |  |  |  |  |  |  |
| 14. Job-Related Traumatic Stress T2 | 1.18 | 0.58 | 0—4 | .49^**^ | .49^**^ | .46^**^ | .36^**^ | .49^**^ | .31^**^ | -.04 | -.14^**^ | -.11^**^ | .64^**^ | .74^**^ | .56^**^ | .79^**^ | – |  |  |  |  |  |  |  |  |  |  |  |  |  |
| 15. Job-Related Traumatic Stress T3 | 1.35 | 0.54 | 0—4 | .47^**^ | .48^**^ | .63^**^ | .39^**^ | .49^**^ | .47^**^ | -.23^**^ | -.25^**^ | -.33^**^ | .67^**^ | .69^**^ | .79^**^ | .67^**^ | .73^**^ | – |  |  |  |  |  |  |  |  |  |  |  |  |
| 16. Job Stress and Burnout Self-Efficacy T1 | 4.78 | 0.82 | 1—7 | -.64^**^ | -.53^**^ | -.48^**^ | -.65^**^ | -.61^**^ | -.51^**^ | .53^**^ | .46^**^ | .50^**^ | -.49^**^ | -.40^**^ | -.33^**^ | -.37^**^ | -.29^**^ | -.25^**^ | – |  |  |  |  |  |  |  |  |  |  |  |
| 17. Job Stress and Burnout Self-Efficacy T2 | 4.86 | 0.64 | 1—7 | -.61^**^ | -.66^**^ | -.51^**^ | -.64^**^ | -.75^**^ | -.60^**^ | .49^**^ | .60^**^ | .53^**^ | -.42^**^ | -.48^**^ | -.38^**^ | -.33^**^ | -.38^**^ | -.34^**^ | .79^**^ | – |  |  |  |  |  |  |  |  |  |  |
| 18. Job Stress and Burnout Self-Efficacy T1 | 4.86 | 0.61 | 1—7 | -.60^**^ | -.60^**^ | -.74^**^ | -.63^**^ | -.63^**^ | -.71^**^ | .43^**^ | .42^**^ | .60^**^ | -.53^**^ | -.52^**^ | -.61^**^ | -.28^**^ | -.32^**^ | -.46^**^ | .74^**^ | .76^**^ | – |  |  |  |  |  |  |  |  |  |
| 19. Social Support Self-Efficacy T1 | 4.86 | 1.19 | 1—7 | -.23^**^ | -.11^**^ | -.13^**^ | -.19^**^ | -.15^**^ | -.12^**^ | .12^**^ | .12^**^ | .25^**^ | -.26^**^ | -.19^**^ | -.24^**^ | -.24^**^ | -.17^**^ | -.27^**^ | .25^**^ | .23^**^ | .12^**^ | – |  |  |  |  |  |  |  |  |
| 20. Social Support Self-Efficacy T2 | 5.04 | 0.72 | 1—7 | -.25^**^ | -.30^**^ | -.21^**^ | -.24^**^ | -.34^**^ | -.24^**^ | .20^**^ | .27^**^ | .30^**^ | -.33^**^ | -.44^**^ | -.41^**^ | -.20^**^ | -.36^**^ | -.37^**^ | .30^**^ | .43^**^ | .31^**^ | .61^**^ | – |  |  |  |  |  |  |  |
| 21. Social Support Self-Efficacy T3 | 5.04 | 0.61 | 1—7 | -.06^*^ | -.05 | -.21^**^ | .02 | -.08^**^ | -.07^*^ | -.08^**^ | -.01 | .08^**^ | -.16^**^ | -.30^**^ | -.31^**^ | -.17^**^ | -.28^**^ | -.38^**^ | .02 | .10^**^ | .19^**^ | .41^**^ | .55^**^ | – |  |  |  |  |  |  |
| 22. Perceived Social Support T1 | 3.05 | 0.69 | 1—5 | -.39^**^ | -.23^**^ | -.29^**^ | -.41^**^ | -.35^**^ | -.30^**^ | .25^**^ | .29^**^ | .23^**^ | -.40^**^ | -.41^**^ | -.34^**^ | -.29^**^ | -.32^**^ | -.30^**^ | .43^**^ | .41^**^ | .34^**^ | .46^**^ | .44^**^ | .50^**^ | – |  |  |  |  |  |
| 23. Perceived Social Support T2 | 3.25 | 0.55 | 1—5 | -.32^**^ | -.32^**^ | -.30^**^ | -.35^**^ | -.42^**^ | -.35^**^ | .21^**^ | .32^**^ | .18^**^ | -.32^**^ | -.45^**^ | -.31^**^ | -.24^**^ | -.37^**^ | -.33^**^ | .32^**^ | .45^**^ | .33^**^ | .38^**^ | .53^**^ | .51^**^ | .88^**^ | – |  |  |  |  |
| 24. Perceived Social Support T3 | 3.19 | 0.56 | 1—5 | -.29^**^ | -.28^**^ | -.43^**^ | -.30^**^ | -.35^**^ | -.36^**^ | .23^**^ | .28^**^ | .27^**^ | -.42^**^ | -.47^**^ | -.45^**^ | -.27^**^ | -.31^**^ | -.45^**^ | .29^**^ | .34^**^ | .41^**^ | .38^**^ | .44^**^ | .64^**^ | .79^**^ | .84^**^ | – |  |  |  |
| 25. Secondary Trauma Exposure T1 | 4.21 | 2.39 | 0—10 | -.09^**^ | -.06^*^ | -.04 | -.04 | -.04 | -.06 | .07^*^ | .04 | .06^*^ | -.03 | -.02 | -.04 | .03 | .02 | -.02 | .09^**^ | .08^**^ | .10^**^ | .00 | .05 | .02 | .03 | .03 | .05 | – |  |  |
| 26. Expectancy of Improvement T1 | 6.05 | 1.61 | 1—9 | -.03 | -.18^**^ | .03 | -.03 | -.16^**^ | -.00 | .09^**^ | .15^**^ | .14^**^ | -.02 | -.17^**^ | -.11^**^ | .05 | -.09^**^ | -.08^**^ | .04 | .17^**^ | .05 | .12^**^ | .31^**^ | .12^**^ | .11^**^ | .12^**^ | .04 | .08^**^ | – |  |
| 27. Perceived Intervention Credibility T1 | 5.17 | 1.68 | 1—9 | -.15^**^ | -.28^**^ | -.07^*^ | -.14^**^ | -.29^**^ | -.16^**^ | .14^**^ | .18^**^ | .17^**^ | -.10^**^ | -.22^**^ | -.12^**^ | .00 | -.13^**^ | -.07^*^ | .15^**^ | .27^**^ | .16^**^ | .10^**^ | .21^**^ | .07^*^ | .13^**^ | .17^**^ | -.06^*^ | .01 | .60^**^ | – |

*Note.* *N* = 1240.; **p* < .05. ***p* < .01.; T1 = measurement at baseline, T2 = measurement at posttest, T3 = measurement at a 6 month follow-up
